# Safety of domperidone in treating nausea associated with dihydroergotamine infusion and headache

**DOI:** 10.1212/WNL.0000000000003429

**Published:** 2016-12-13

**Authors:** Nathaniel M. Robbins, Hiroyuki Ito, Melvin M. Scheinman, Peter J. Goadsby

**Affiliations:** From the Department of Neurology (N.M.R.), Dartmouth-Hitchcock Medical Center, Lebanon, NH; Section of Cardiac Electrophysiology, Division of Cardiology (H.I., M.M.S.), and Department of Neurology (P.J.G.), University of California, San Francisco; and NIHR-Wellcome Trust King's Clinical Research Facility (P.J.G.), King's College London, UK.

## Abstract

**Objective:**

To determine the safety of domperidone in the treatment of nausea associated with dihydroergotamine (DHE) infusion and headache.

**Methods:**

We audited our use of domperidone for the inpatient management of nausea, focusing on known safety concerns, particularly potential cardiac arrhythmias.

**Results:**

We reviewed 103 consecutive admissions of 90 patients admitted for IV DHE by infusion. Most admissions were to treat chronic migraine with (n = 53) or without (n = 46) aura. Domperidone was administered in 85 of 103 encounters and was well-tolerated at doses up to 80 mg/d. A significant side effect, akathisia, was observed in one patient. Baseline ECG with corrected QT interval (QTc) was obtained on all patients. Repeat ECG after domperidone was obtained in 21 patients, whose baseline characteristics did not differ from the group as a whole. ECG was interpreted blindly by a cardiac electrophysiologist. QTc did not differ before and after domperidone administration (Wilcoxon signed-rank test, median [interquartile range] 435.0 [410.5–453.0] at admission and 427.0 [399.0–452.5] after domperidone; *p* = 0.15). In combination with other antiemetics, domperidone was effective in treating nausea such that no patients had refractory nausea severe enough to limit DHE dose.

**Conclusions:**

This retrospective audit demonstrates that domperidone is safe in the treatment of nausea associated with inpatient DHE infusion and headache. While larger prospective trials are necessary to confirm these results and assess efficacy, current evidence and clinical experience suggests that domperidone is safe and useful for nausea and headache management.

**Classification of evidence:**

This study provides Class IV evidence that for patients with headache undergoing DHE infusion, domperidone is safe and effective in the treatment of nausea.

Migraine is the sixth leading cause of years of life lost to disability worldwide.^1^ Patients with chronic migraine can benefit from inpatient treatment to reduce the severity and frequency of headache. For refractory migraine, IV dihydroergotamine (DHE) has been a cornerstone of treatment for over 2 decades.^2,3^ Nausea is a common side effect of DHE administration and can be severe and dose-limiting.

Previously we showed that nausea control is central to maximizing DHE dosing and improving headache control.^2^ Nausea treatment often relies on antidopaminergic agents such as metoclopramide, chlorpromazine, and prochlorperazine, but these medications can cause serious side effects such as akathisia, sedation, extrapyramidal disorders including acute dystonic reactions, corrected QT interval (QTc) prolongation, and torsade de pointes.

Domperidone is a less expensive alternative that, in our clinical experience, is often more easily tolerated. In our headache practice, we routinely premedicate patients admitted for IV DHE with domperidone in order to prevent nausea. Domperidone is a peripherally acting dopamine antagonist widely used for decades to treat gastric motility disorders and nausea.^4^ Despite its ascribed peripheral mechanism of action, high-quality studies have shown that domperidone is safe and efficacious in treating migraine.^5^ It is not approved in the United States, however, due to concerns for cardiac arrhythmogenicity stemming from preclinical data, case reports, and retrospective epidemiologic studies.^6^ Deaths have been restricted to those patients receiving high IV doses, those with exacerbating conditions such as hypokalemia, or those taking other medications that prolong QTc or interfere with metabolism.^4,7,8^ Prospective trials have not found a clinically significant risk of death.^9^ Supporting domperidone's safety, millions of prescriptions have been written in Canada and only 18 possible cardiac events have been reported, and no deaths.^7^

In the current study, we review our experience using domperidone at the University of California, San Francisco (UCSF) Headache Center. Our primary objective was to assess the safety of domperidone in the inpatient treatment of headache and DHE-associated nausea, with a focus on common and dangerous side effects including cardiac arrhythmias.

## METHODS

The experience of adults admitted for IV DHE treatment to the UCSF Headache Center between July 2012 and May 2015 was audited. Treatment was at the discretion of the attending neurologist. In our practice, we routinely use domperidone 10 mg orally prior to DHE infusion, with increased dosing as needed for persistent nausea. All patients who received at least one dose of domperidone were included. Patients with at least one ECG after exposure to domperidone were included in the analysis of cardiac safety.

### Standard protocol approvals, registrations, and patient consents.

This study was approved by the UCSF Committee on Human Research. Patient consent was waived for this retrospective audit.

### Nausea severity.

Nausea severity was defined as absent (no as-needed antiemetics), mild (some as-needed antiemetics but no increase in scheduled dosing), moderate (aprepitant added or increased, or additional antiemetics required despite maximum aprepitant), severe (DHE infusion slowed despite maximum antiemetics), or refractory (DHE stopped due to nausea).

### QT interval measurement.

Admission ECG QT and QTc were interpreted automatically at the time of the admission. All ECGs for patients included in the QTc analyses were also manually interpreted in a blinded fashion by 2 cardiac electrophysiologists (M.M.S., H.I.). If patients had multiple ECGs on domperidone, QTc was averaged for comparison.

### Statistical methods.

All tests were done with IBM (Armonk, NY) SPSS Statistics v22. We compared manually interpreted QTc before and after domperidone administration using the Wilcoxon signed-rank test with 2-tailed significance. A drug-induced QTc prolongation of ≥30 ms has been proposed as the cutoff for defining a clinically significant change.^10^ To increase sensitivity for detecting safety concerns, we used a postdomperidone increase of ≥10 ms. Assuming a 10-ms difference and a common SD of 15 ms, we calculated that 26 patients would be required to achieve adequate power. We compared groups using Pearson χ^2^, Mann-Whitney *U*, or Fisher exact test as appropriate.

## RESULTS

We admitted 119 patients to the UCSF Headache Center between July 2012 and May 2015. Ninety unique patients underwent 103 admissions for DHE. Of the 90 patients, 89 received at least one DHE dose. Most patients had chronic migraine with or without aura. Eleven had posttraumatic headache of a migrainous type, 26 had medication overuse at admission, and 10 had new daily persistent headache. Six had other diagnoses: cluster headache, nummular headache, hypnic headache, and hemicrania continua. Domperidone was administered in 82/103 patient encounters to 74/90 patients. See table 1 for baseline demographic and clinical characteristics of the entire sample and the subset that received domperidone.

**Table 1:**
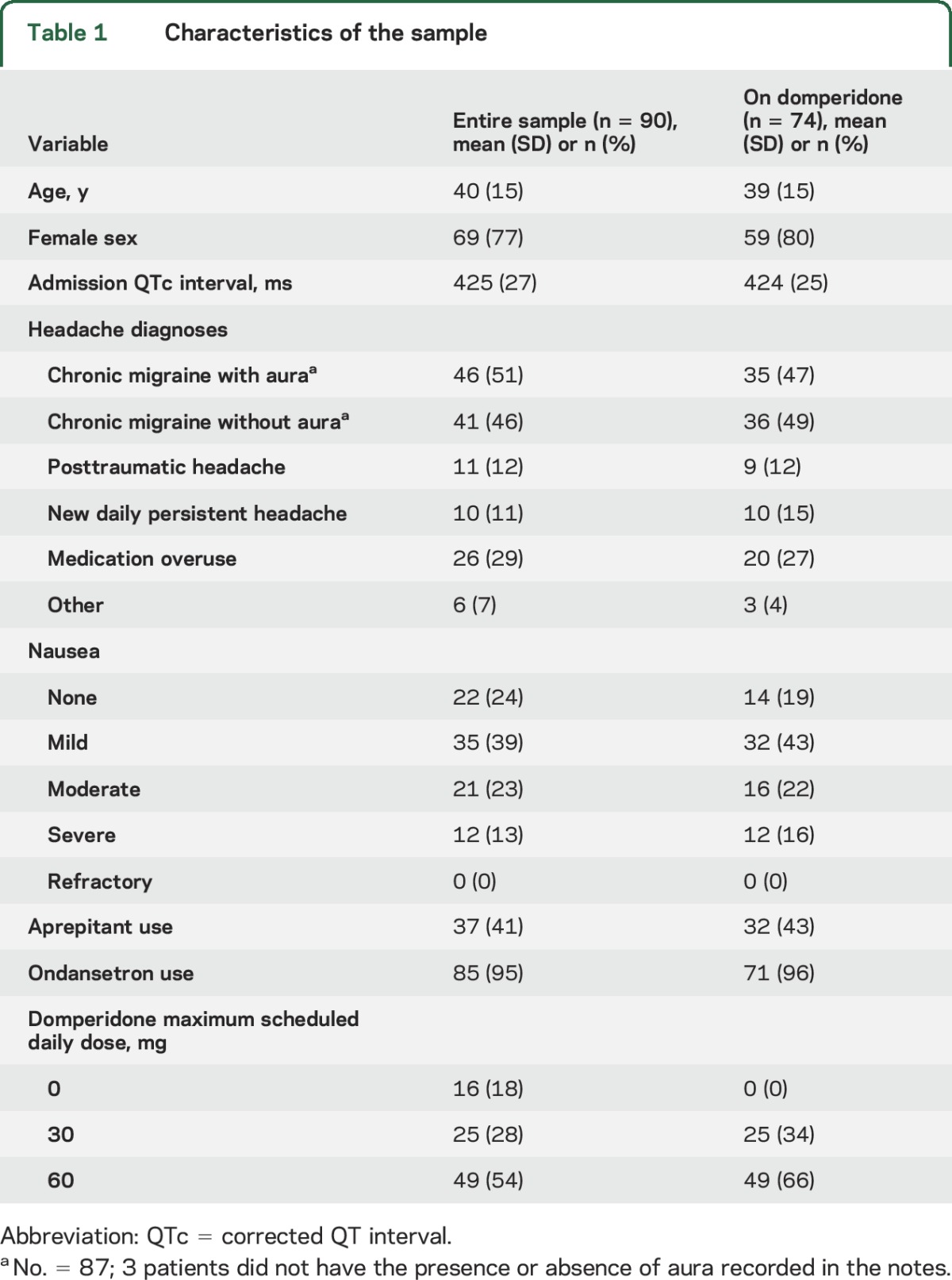
Characteristics of the sample

### Safety of domperidone.

Daily domperidone dose ranged from 30 to 80 mg. Domperidone was withheld in one patient for long admission QTc (498 ms), and in another patient because of cardiac history. One patient was transferred prior to first dose due to concern for stroke. The other 13 patients with 18 admissions took no domperidone due to patient refusal or an inability to obtain the medication.

Nausea, diarrhea, increased headache, chest pressure, and leg and abdominal cramping were common in this sample, but these are anticipated consequences of DHE infusion^2^ and not attributable to domperidone. Possible adverse events were observed in 2/82 encounters. One patient experienced akathisia, which was probably attributed to domperidone. Another patient developed epigastric pain and transaminitis and was diagnosed with acalculous cholecystitis requiring cholecystectomy. Domperidone use seemed unrelated—pathology showed chronic inflammation and the patient confirmed the chronicity clinically.

### Cardiac safety.

Twenty-one patients underwent repeat ECGs on domperidone for the following indications: chest pain (n = 5), epigastric or abdominal pain (n = 3), QTc monitoring (n = 1), unknown (n = 3), or exit QTc screening (n = 9). See table 2 for a comparison of this group with the rest of the study population.

**Table 2:**
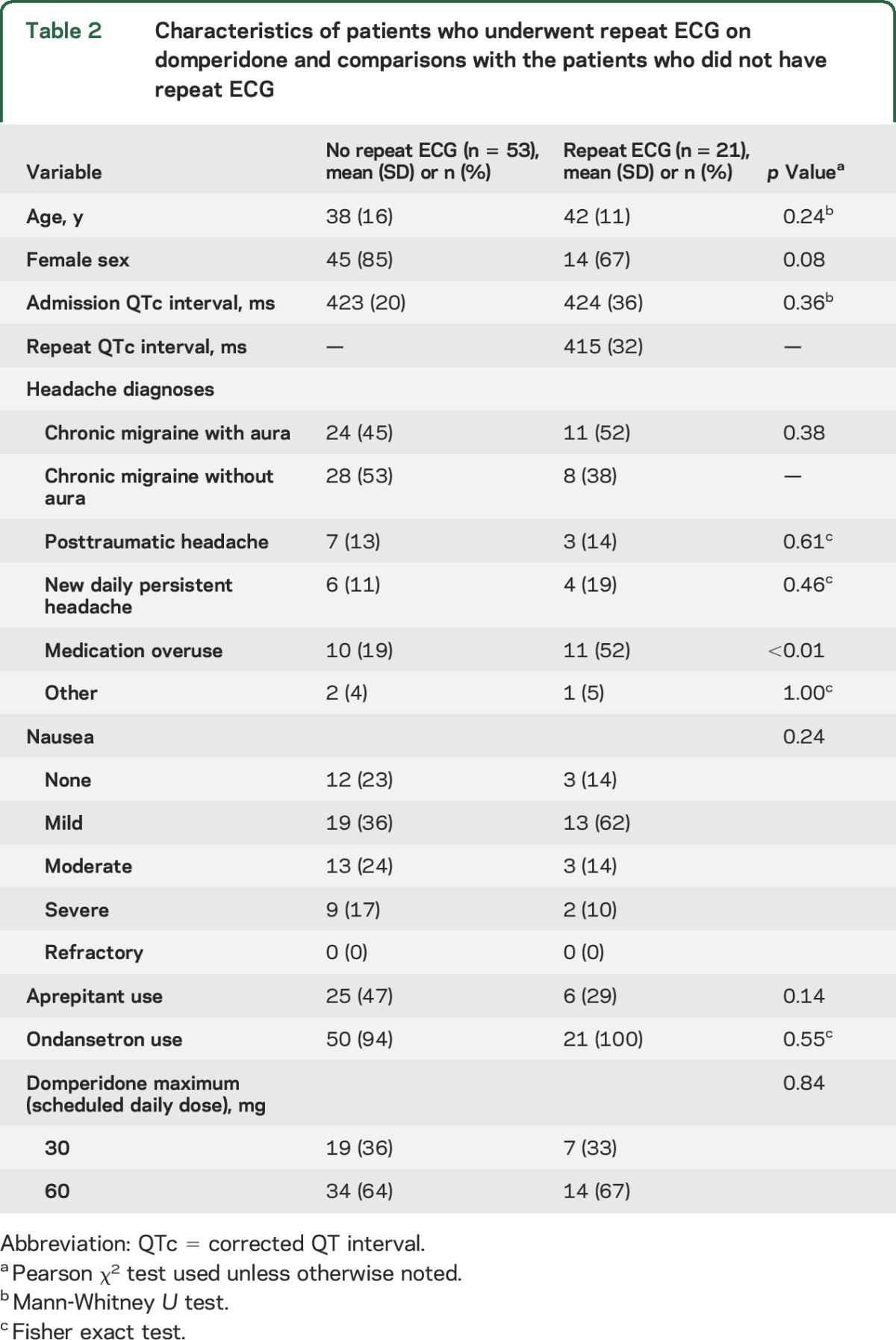
Characteristics of patients who underwent repeat ECG on domperidone and comparisons with the patients who did not have repeat ECG

Domperidone did not have a statistically or clinically meaningful effect on QTc (Wilcoxon signed-rank test, median [interquartile range] 435.0 [410.5–453.0] at admission and 427.0 [399.0–452.5] after domperidone; *z* = −1.44, *p* = 0.15). See figure for a comparison of QTc in patients who received an ECG before and after domperidone administration.

**Figure:**
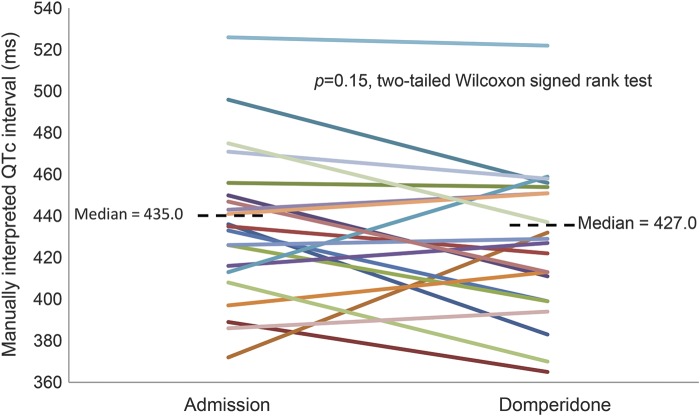
Corrected QT interval (QTc) before and after domperidone administration by participant (n = 21)

To ensure consistency between manual and automated QTc interpretation, we used 2-way mixed, single-measures intraclass correlation coefficients (ICC) using an absolute agreement definition. The resultant ICC was excellent (ICC 0.81, 95% confidence interval 0.59–0.91).

## DISCUSSION

In this population, we observed no serious side effects or QTc prolongation. We also found that domperidone helps manage DHE-associated nausea in combination with other antiemetics. These findings align with decades of internationally accumulated clinical experience and numerous prospective trials. Although a range of options exist to treat migraine, migraine-associated nausea, and emergent nausea with DHE, most treatments are dangerous or replace nausea with disabling sedation. As a less sedating alternative, domperidone is invaluable in migraine management given that safety concerns are allayed.

Limitations of this study include its retrospective design and small sample size. Efficacy, including comparisons with centrally acting dopaminergic blockers, could not be assessed. Rare events and conditions, such as a concealed long QT syndrome, may be missed. In addition, this population was relatively young and healthy, and few patients had exacerbating conditions, such as electrolyte disorders or coadministration of QT prolonging agents. Finally, side effects were not specifically queried; we relied on progress notes and discharge summaries. Minor and asymptomatic side effects, such as liver enzyme or prolactin elevation, may have been missed.

It should be noted that no patients received high domperidone doses, and that all patients had baseline screening ECGs. While caution should be used when treating patients with risk factors such as older age, heart disease, concurrent use of QT prolonging medications, and hypokalemia, current evidence suggests that domperidone is safe and effective in the vast majority of individuals.

This audit provides Class IV evidence that domperidone is safe for treating nausea associated with DHE infusion and headache in a tertiary hospital. Future studies should extend these findings to other settings (e.g., outpatient clinics) and other populations (e.g., nonmigrainous headache disorders, patients with risk factors). Given domperidone's effectiveness and tolerability, concern for safety should not preclude its use in a well-screened headache population.

## GLOSSARY

DHE: dihydroergotamine
ICC: intraclass correlation coefficient
QTc: corrected QT interval
UCSF: University of California, San Francisco

## References

[1] Vos T, Flaxman AD, Naghavi M, et al. Years lived with disability (YLDs) for 1160 sequelae of 289 diseases and injuries 1990–2010: a systematic analysis for the Global Burden of Disease Study. Lancet 2010;380:2163–2196. 10.1016/S0140-6736(12)61729-2 PMC6350784 23245607

[2] Nagy AJ, Gandhi S, Bhola R, Goadsby PJ. Intravenous dihydroergotamine for inpatient management of refractory primary headaches. Neurology 2011;77:1827–1832. 22049203 10.1212/WNL.0b013e3182377dbb

[3] Raskin NH. Repetitive intravenous dihydroergotamine as therapy for intractable migraine. Neurology 1986;36:995. 3520384 10.1212/wnl.36.7.995

[4] Ahmad N, Keith-Ferris J, Gooden E, Abell T. Making a case for domperidone in the treatment of gastrointestinal motility disorders. Curr Opin Pharmacol 2006;6:571–576. 16997628 10.1016/j.coph.2006.07.004

[5] Dowson A, Ball K, Haworth D. Comparison of a fixed combination of domperidone and paracetamol (domperamol) with sumatriptan 50 mg in moderate to severe migraine: a Randomised UK Primary Care Study. Curr Med Res Opin 2000;16:190–197. 11191009

[6] van Noord C, Eijgelsheim M, Stricker BHC. Drug- and non-drug-associated QT interval prolongation. Br J Clin Pharmacol 2010;70:16–23. 20642543 10.1111/j.1365-2125.2010.03660.x PMC2909803

[7] Health Canada. Summary Safety Review: Domperidone: Serious Abnormal Heart Rhythms and Sudden Death (Cardiac Arrest). Health Canada; 2015.

[8] Buffery PJ, Strother RM. Domperidone safety: a mini-review of the science of QT prolongation and clinical implications of recent global regulatory recommendations. NZ Med J 2015;128:66–74. 26117678

[9] Djeddi D, Kongolo G, Lefaix C, Mounard J, Léké A. Effect of domperidone on QT interval in neonates. J Pediatr 2008;153:663–666. 18589449 10.1016/j.jpeds.2008.05.013

[10] The European Agency for the Evaluation of Medicinal Products Committee for Proprietary Medicinal Products. The Assessment of the Potential for Qt Interval Prolongation by Non-cardiovascular Medicinal Products. London: Committee for Proprietary Medicinal Products; 1997:986–996.

